# Polymorphisms in Gag spacer peptide 1 confer varying levels of resistance to the HIV- 1maturation inhibitor bevirimat

**DOI:** 10.1186/1742-4690-7-36

**Published:** 2010-04-20

**Authors:** Catherine S Adamson, Michael Sakalian, Karl Salzwedel, Eric O Freed

**Affiliations:** 1 Virus-Cell Interaction Section, HIV Drug Resistance Program, National Cancer Institute, Frederick, MD 21702-1201, USA; 2 Panacos Pharmaceuticals Inc, 209 Perry Parkway, Gaithersburg, MD 20877, USA; 3 Bute Medical School, University of St Andrews, Westburn Lane, St Andrews, Fife, KY16 9TS, UK

## Abstract

**Background:**

The maturation inhibitor bevirimat (BVM) potently inhibits human immunodeficiency virus type 1 (HIV-1) replication by blocking capsid-spacer peptide 1 (CA-SP1) cleavage. Recent clinical trials demonstrated that a significant proportion of HIV-1-infected patients do not respond to BVM. A patient's failure to respond correlated with baseline polymorphisms at SP1 residues 6-8.

**Results:**

In this study, we demonstrate that varying levels of BVM resistance are associated with point mutations at these residues. BVM susceptibility was maintained by SP1-Q6A, -Q6H and -T8A mutations. However, an SP1-V7A mutation conferred high-level BVM resistance, and SP1-V7M and T8Δ mutations conferred intermediate levels of BVM resistance.

**Conclusions:**

Future exploitation of the CA-SP1 cleavage site as an antiretroviral drug target will need to overcome the baseline variability in the SP1 region of Gag.

## Background

Human immunodeficiency virus type 1 (HIV-1) infectivity is dependent on virion maturation, a morphological rearrangement of the viral core that occurs concomitant with virus particle release [1,2]. HIV-1 maturation is triggered by cleavage of the Gag polyprotein, catalyzed by the viral protease (PR), into the matrix (MA), capsid (CA), spacer peptide 1 (SP1), nucleocapsid (NC), spacer peptide (SP2) and p6 constituents. Gag cleavage occurs as a sequential cascade of steps that is kinetically controlled by the differential rate of processing at each of the five cleavage sites in Gag [3-9]. First, Gag is cleaved into two fragments, MA-CA-SP1 and NC-SP2-p6. Next, the MA and p6 domains are released, and finally the CA and NC domains are liberated from the remaining CA-SP1 and NC-SP2 processing intermediates. Morphological rearrangement of the viral core is triggered by the release of the mature CA domain, which reassembles into a hexameric lattice to form a condensed conical core.

The small molecule 3-*O*-(3',3'-dimethylsuccinyl)-betulinic acid (DSB), also known as PA-457, MPC-4326, or bevirimat (BVM), potently inhibits HIV-1 replication by inhibiting a late step in the proteolytic processing cascade of Gag by specifically blocking the cleavage of SP1 from the C-terminus of CA [10-12]. Inhibiting CA-SP1 cleavage results in the formation of aberrant, non-infectious particles that fail to undergo proper maturation [9,10]. The novel mechanism of action of BVM led to its designation as the first in a new class of antiretroviral drugs known as maturation inhibitors [1,13,14].

The potent *in vitro *activity of BVM [10], together with promising pharmacological and safety profiles in animal models and phase I clinical trials [15-18], led to clinical testing of BVM efficacy in HIV-1-infected patients. Initial phase II clinical trials reported statistically significant, dose-dependent viral load reductions in HIV-1-infected patients [19]. However, further studies showed that up to 50% of patients receiving BVM did not exhibit significant viral load reductions [20]. Optimal BVM plasma concentrations were observed in many of the non-responder patients, implying that virological parameters could be responsible for part of the observed variable clinical outcome [20]. Population genotyping of patient isolates demonstrated that the BVM-resistance mutations identified in *in vitro *selection studies were not present in the non-responding cohort [21]. The *in vitro *selected BVM-resistance mutations map to three highly conserved residues at the extreme C-terminus of CA (CA-H226Y, L231M and L231F) and the first and third residues of SP1 (SP1-A1V, A3V and A3T) [10,11,22]. Instead, the presence of baseline polymorphisms at SP1 residues 6-8 in the relatively non-conserved C-terminal portion of SP1 correlated with patients' failure to respond [20]. Patients infected with isolates encoding the clade B consensus amino acid sequence glutamine-valine-threonine (QVT) at these positions were significantly more likely to respond to BVM treatment than were patients infected with virus encoding polymorphisms at these positions [20].

A high-throughput *in vitro *phenotypic infectivity assay has been used to evaluate the correlation between a panel of naturally occurring Gag polymorphisms at SP1 residues 6-8 (SP1/6-8) and BVM susceptibility [23]. Specific polymorphisms at SP1 residues 7 and 8 (SP1-V7A, -V7M, -T8Δ and -T8N) were shown to be sufficient to confer decreased BVM susceptibility, while other mutations at SP1 residues 6 and 8 (SP1-Q6A, -Q6H and -T8A) retained sensitivity [23]. BVM susceptibility was reported as a fold change in IC_50 _value; the impact of these mutations on CA-SP1 processing or replication fitness was not reported.

## Results and Discussion

### Effect of SP1 mutations on CA-SP1 processing

**Figure 1:**
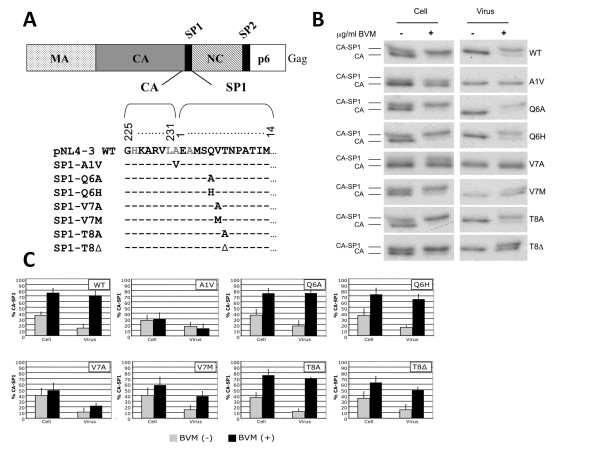
**Mutagenesis at SP1 residues 6-8 results in varying degrees of CA-SP1 processing in the presence of BVM**. (A) Mutagenesis of SP1 residues 6-8. HIV-1 Gag is represented at the top. The MA, CA, NC and p6 domains and the SP1 and SP2 spacer peptides are indicated. The alignment shows the pNL4-3 wild type (WT) amino acid sequence at the CA-SP1 junction in Gag and the panel of SP1 mutant derivatives examined in this study. The residues to which BVM resistance was previously mapped *in vitro *are shaded in grey. (B and C) Quantitative CA-SP1 processing assay. HeLa cells were transfected with WT pNL4-3 and the panel of SP1 mutant derivatives and cultured either without BVM or in 1 μg/ml BVM. Cells were metabolically labeled for 2 h with [^35^S]Met/Cys, and released virions were pelleted by ultracentrifugation. Cell and virus lysates were immunoprecipitated with HIV-Ig, and processing of CA-SP1 to CA was analyzed by SDS-PAGE and fluorography (B) and by phosphorimager analysis to quantify the percentage of CA-SP1 relative to total CA-SP1 plus CA (C). Error bars indicate standard deviations (n = 3-5).

To extend our understanding of the relationship between Gag polymorphisms at SP1/6-8 and BVM susceptibility, we employed a quantitative biochemical CA-SP1 processing assay that we have previously used to analyze *in vitro*-selected BVM-resistance mutations [22,24]. Point mutations at SP1/6-8 were introduced into the infectious HIV-1 molecular clone pNL4-3 [25] to generate pNL4-3 SP1-Q6A, -Q6H, -V7A, -V7 M, -T8A and -T8Δ (Fig. 1A). This panel includes both alanine-scanning mutations across the residues of interest and several observed Gag polymorphisms (SP1-Q6H, -V7A, -V7 M, -T8A, and -T8Δ) [23,26]. WT pNL4-3, which contains the QVT motif at SP1/6-8, was used as a BVM-susceptible virus and the previously characterized SP1-A1V mutant served as a prototypical BVM-resistant virus [10,16,22,24]. The CA-SP1 processing assay was performed as previously described [22,24,27]. Briefly, HeLa cells transfected with WT or mutant pNL4-3 molecular clones were cultured either with no drug or with 1 μg/ml BVM. The cells were metabolically labeled with [^35^S]Met/Cys, and cell- and virion-associated proteins were immunoprecipitated with HIV-Ig. CA-SP1 cleavage was detected (Fig. 1B) and quantified as the percentage of CA-SP1 relative to total CA-SP1 plus CA (Fig. 1C). Consistent with our previous results, treatment of WT-transfected cells with BVM resulted in a marked accumulation of CA-SP1 in both cell and virion fractions. Similar levels of CA-SP1 were observed in the SP1-Q6A, -Q6H and -T8A BVM-treated samples. In contrast, CA-SP1 processing in SP1-V7A BVM-treated samples was similar to that seen in untreated WT or SP1-A1V samples. Interestingly, intermediate levels of CA-SP1 processing were observed in SP1-V7M and -T8Δ virions produced from BVM-treated cells. The result with SP1-T8Δ is consistent with previous analysis of this mutant [28]. The biochemical data obtained were also used to estimate virus release efficiency for each of the SP1 mutant derivatives in the absence and presence of BVM relative to untreated WT. Virus release efficiency was not significantly affected, with the exception of the SP1-V7A mutant cultured in the absence of drug, where a 3-fold reduction in virus release efficiency was observed (data not shown).

### Effect of SP1 mutations on sensitivity to BVM in a single-cycle infectivity assay

**Figure 2:**
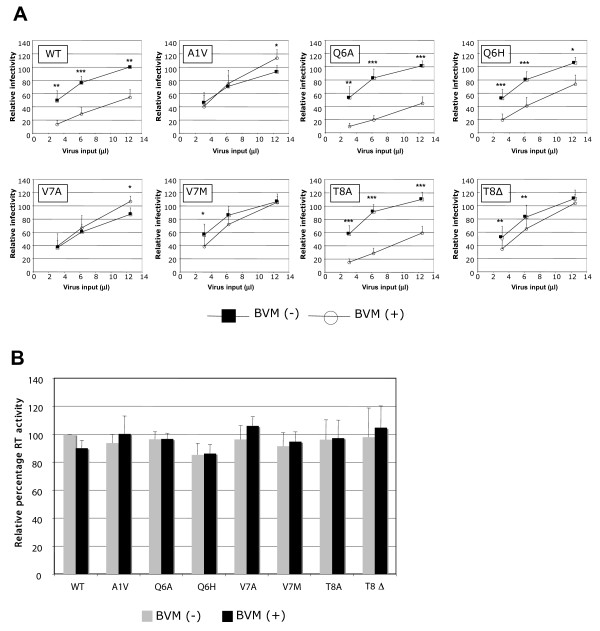
**Residue 6-8 mutations result in varying levels of resistance to BVM**. (A) Virus stocks produced from HeLa cells either in the absence of BVM or in the presence of 1 μg/ml BVM were used to infect the TZM-bl indicator cell line. Infectivity was measured 48 h postinfection by determining levels of luciferase activity. Relative infectivity was calculated by normalization of the untreated WT virus at the 12.5 μl viral input to 100%. Paired t tests were performed to evaluate differences between means. Statistically significant differences between pairs of means are indicated (*** P = 0.0001, ** P = 0.001, * = 0.01). (B) Virus inputs were verified by confirming that virus stocks contained comparable RT activities. All data shown are means and standard deviations from three independent experiments, performed in duplicate.

We next sought to examine the effect of the SP1/6-8 mutations on virus infectivity using a single-cycle infectivity assay in the TZM-bl indicator cell line [29,30]. Virions produced from transfected HeLa cells cultured either without drug or with 1 μg/ml BVM were used to infect TZM-bl cells and luciferase activity was measured 48 hours postinfection (Fig. 2). The SP1/6-8 mutations did not significantly impact virus infectivity when particles were generated in the absence of BVM. However, virus infectivity was clearly inhibited when the WT, SP1-Q6A, -Q6H and -T8A viruses were generated in the presence of BVM. In contrast, BVM treatment did not reduce the ability of SP1-A1V or -V7A viruses to infect TZM-bl cells. Infectivity of viruses harboring the SP1-V7M and -T8Δ mutations was only moderately inhibited when produced in the presence of BVM.

### Effect of SP1 mutations on sensitivity to BVM in the context of a spreading infection

**Figure 3:**
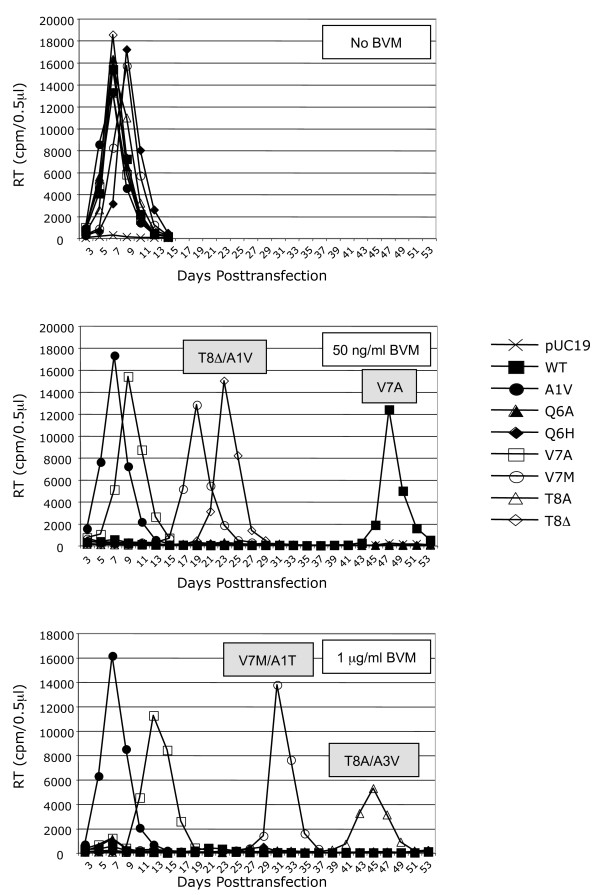
**Replication kinetics of viruses containing mutations in SP1 residues 6-8**. Jurkat T cells were transfected with WT pNL4-3 and the panel of SP1 mutant derivatives and cultured either without BVM or in 50 ng/ml or 1 μg/ml BVM. Cells were split every 2 days, and supernatants were reserved at each time point for RT analysis. All originally introduced mutations were maintained. The grey boxes indicate those cultures in which an additional mutation is acquired; both the introduced and the acquired mutations are indicated. The results shown are representative of at least 2 independent experiments. Results from the repeat experiment are described in the text.

The biochemical and single-cycle infectivity data presented above suggest that the SP1/6-8 mutations confer varying levels of BVM resistance without compromising virus infectivity. To confirm this result and determine the effects of these mutations on virus replication capacity, we evaluated virus replication kinetics in the Jurkat T cell line. Each construct was transfected into the Jurkat T-cell line, and the cells were cultured either without BVM or in the presence of a suboptimal (50 ng/ml) or inhibitory (1 μg/ml) drug concentration (Fig. 3). Virus replication was monitored by RT activity. The maintenance of existing and/or acquisition of additional mutations was determined by extracting genomic DNA from cells at the peak of RT activity, PCR amplification of the Gag and PR coding regions and DNA sequencing [22,24,27] (Fig. 3). WT virus was fully inhibited at 1 μg/ml BVM but developed BVM resistance after 47 days in the presence of 50 ng/ml BVM by acquisition of an SP1-V7A mutation. The SP1-V7A substitution has not been observed in previous *in vitro *BVM-resistance selection studies [10,11,22,24]. In agreement with our previous studies, the SP1-A1V mutant was fully resistant to BVM as it replicated with WT kinetics independent of BVM concentration and did not acquire additional mutations. The SP1/6-8 mutations were maintained in all cultures in which detectable virus replication occurred. In the absence of BVM, the mutant viruses replicated with essentially WT kinetics and did not acquire additional amino acid substitutions, demonstrating that no significant fitness cost was associated with the SP1/6-8 mutations in the Jurkat cell system. The SP1-V7A mutation exhibited resistance to BVM as its replication was only moderately delayed with increasing BVM concentration and it replicated without acquiring additional mutations. At the 50 ng/ml BVM concentration, the SP1-V7M virus was capable of replicating, albeit with a significant delay, without acquiring additional amino acid substitutions. However, at the 1 μg/ml concentration, detectable replication was even further delayed and was accompanied by the acquisition of an SP1-A1T substitution (Fig. 3). Although the SP1-A1T change has not previously been reported in association with BVM resistance [10,11,22,24], it maps to the same residue as the robust SP1-A1V BVM-resistance mutation. In a repeat experiment, the same pattern of replication and mutation acquisition was observed for the V7M mutant, except that at 1 μg/ml BVM a CA-V230I substitution and the previously characterized SP1-A3V mutation were detected when virus replication emerged after 29 days in culture (data not shown). The CA-V230I substitution has previously been reported to be acquired in the context of a CA-L231 MBVM-resistance mutation combined with a mutant PR when propagated in the presence of BVM [24]. Interestingly, the CA-V230I substitution occurs frequently in HIV-1 isolates [26,31,32] and therefore could represent an additional Gag polymorphism associated with BVM resistance. Replication of SP1-T8Δ was significantly delayed at the 50 ng/ml BVM concentration and was only detected upon acquisition of the SP1-A1V BVM-resistance mutation (Fig. 3). No detectable replication was observed for the SP1-T8Δ virus at 1 μg/ml BVM for up to 53 days in culture. However, in a repeat experiment, replication emerged after 68 days in culture and was accompanied by acquisition of the SP1-A3V mutation (data not shown). No replication was detectable for the SP1-Q6A and -Q6H mutants in the presence of either 50 ng/ml or 1 μg/ml BVM. The SP1-T8A virus did not replicate at 50 ng/ml BVM; however, replication of this mutant was detected at 1 μg/ml BVM at day 45 posttransfection and was accompanied by acquisition of the SP1-A3V mutation (Fig. 3).

## Conclusions

The data presented here demonstrate that point mutations corresponding to commonly observed polymorphisms at SP1 residues 6-8 in HIV-1 clinical isolates confer varying degrees of susceptibility to BVM in the NL4-3 background. The SP1-Q6A, Q6H, and T8A mutants retained complete sensitivity to BVM. Mutations SP1-V7M and T8Δ exhibited an intermediate level of BVM susceptibility. The SP1-V7M mutant appeared less susceptible to BVM than did SP1-T8Δ; SP1-V7M BVM-treated virions accumulated less unprocessed CA-SP1 and the SP1-V7M virus replicated without acquisition of additional mutations at 50 ng/ml BVM, whereas robust SP1-T8Δ replication required acquisition of a previously characterized BVM-resistance mutation. The SP1-V7A mutant displayed full resistance to BVM in terms of CA-SP1 processing, single-cycle infectivity, and virus replication assays, and its resistance was comparable to that of the robust and frequently observed BVM-resistance mutant SP1-A1V.

The observed variability in BVM susceptibility conferred by different mutations at SP1 residues T8 and V7 is likely due to differential effects on the putative BVM binding pocket at the CA-SP1 junction of Gag. For example, deletion of residue 8 is a more substantial change than a T-to-A substitution and could therefore be predicted to have a greater effect on BVM's ability to bind Gag and exert its antiviral affect. Hence the T8Δ mutant exhibits intermediate resistance to BVM while T8A retains BVM susceptibility. Although binding of BVM to the CA-SP1 junction has previously been suggested by biochemical studies that examined other BVM-resistant mutants [33,34], the lack of high-resolution structural information on this region of Gag hinders further elucidation at this time.

The data obtained in this study are in close agreement with the previously reported high-throughput phenotypic assay used to evaluate the association between Gag polymorphisms at SP1/6-8 and BVM susceptibility [23]. However, we extend the prior findings by evaluating the effects of SP1/6-8 mutations on CA-SP1 processing in the presence and absence of BVM, measuring the effects of these mutations on viral replication capacity, and performing BVM selections analyses with these mutants. In conclusion, baseline polymorphisms in the non-conserved C-terminal portion of SP1 represent a considerable challenge to the clinical development of BVM. In particular, the SP1-V7A polymorphism constitutes a significant obstacle as it displayed robust BVM-resistance in *in vitro *assays and has been shown to occur at high frequency in some HIV-1 subtypes [23]. Future exploitation of the CA-SP1 cleavage site as a molecular target for antiretroviral drug development will need to account for the baseline amino acid variability in this region of Gag.

## Methods

### BVM and cell culture

BVM was prepared as described previously [35] and used at the concentrations indicated. HeLa cells were maintained in Dulbecco Modified Eagle Medium (DMEM) supplemented with 5% (vol/vol) fetal bovine serum (FBS). TZM-bl indicator cells [obtained through the NIH AIDS Research and Reference Reagent Program, Division of AIDS, NIAID; [[29,30]] were maintained in DMEM supplemented with 10% (vol/vol) FBS. The Jurkat T-cell line was maintained in RPMI-1640 supplemented with 10% (vol/vol) FBS. All media were supplemented with L-glutamine (2 mM), penicillin and streptomycin.

### Generation of SP1 mutants and transfections

Point mutations at SP1 residues 6-8 (SP1/6-8) were introduced into the infectious HIV-1 molecular clone pNL4-3 [25] by using site-directed mutagenesis to generate pNL4-3 SP1-Q6A, Q6H, V7A, V7 M, T8A and T8Δ (Fig. 1A). Plasmid DNA was purified with the plasmid purification maxiprep kit (QIAGEN), adjusted to 1 μg/μl and the identities of all plasmids were confirmed by DNA sequencing. HeLa cells were transfected with Lipofectamine 2000 (Invitrogen) according to the manufacturer's instructions. Jurkat T cells were transfected by using DEAE-dextran [36].

### Radioimmunoprecipitation analysis

Methods used for metabolic labeling of HeLa cells, preparation of cell and virus lysates, and immunoprecipitation have been previously described in detail [22,27,37]. Briefly, media and solutions containing BVM at the indicated concentrations were prepared immediately before use and mixed by vortexing. BVM was maintained throughout the transfection and radioimmunoprecipitation procedures. HeLa cells were transfected with wild-type (WT) or the SP1 mutant derivative pNL4-3 clones. Transfected HeLa cells were starved in Cys/Met-free medium for 30 min and then metabolically radiolabeled for 2 h with [^35^S]Cys/Met Pro-mix (Amersham). Virions were pelleted by ultracentrifugation. Cell and virus lysates were immunoprecipitated with pooled immunoglobulin from HIV-1-infected patients (HIV-Ig) obtained through the NIH AIDS Research and Reference Reagent Program, Division of AIDS, NIAID. The radioimmunoprecipitated proteins were separated by sodium dodecyl sulfate-polyacrylamide gel electrophoresis (SDS-PAGE) and exposed to X-ray film and a phosphorimager plate (Fuji) and the bands were quantified by using Quantity One software (Bio-Rad).

### Single-cycle infectivity assay

Virus stocks of were produced by transfecting HeLa cells with WT and SP1 mutant pNL4-3 derivatives and then cultured either without BVM or with 1 μg/ml BVM. Twenty-four hours posttransfection, the cells were washed twice and fresh medium containing either no BVM or 1 μg/ml BVM was added. Following a 4-hour incubation, virus-containing supernatant was collected, filtered and used to infect TZM-bl cells. Infections were carried out for 2 hours in the presence of 10 μg/ml DEAE-dextran. The cells were then washed, cultured without BVM, and luciferase activity measured 48 hours postinfection using the luciferase assay system (Promega). Reverse transcriptase (RT) activities in the virus stocks were measured as previously described [27].

### Replication kinetics

Jurkat T cells were transfected with pNL4-3 WT and SP1 mutant derivatives. BVM was added at the time of transfection at the indicated concentrations and was maintained throughout the course of the experiment. The Jurkat cells were split every two days, supernatant collected at each time point and viral replication monitored by RT activity as previously described [27]. Cell pellets and virus supernatants were harvested on the days of peak RT activity. To verify that the SP1 mutations were maintained and to investigate acquisition of additional mutations, genomic DNA was extracted from cells on the day of peak RT activity using a whole-blood DNA extraction kit (QIAGEN). The entire Gag-PR coding region was then amplified by PCR by using the forward and reverse primers NL516F (5'-TGC CCG TCT GTT GTG TGA CTC-3') and NL2897R (5'-AAA ATA TGC ATC GCC CAC AT-3') respectively. The resultant 2.3 kb PCR product was purified by using the QIAquick PCR purification kit (QIAGEN) and sequenced using the primers NL1410F (5'-GGA AGC TGC AGA ATG GGA TA-3'), NL1754F (5'-TGG TCC AAA ATG CGA ACC-3') and NL2135F (5'-TTC AGA GCA GAC CAG AGC CAA-3') [22,24].

## Competing interests

The authors declare that they have no competing interests.

## Authors' contributions

CSA acquired the data, performed analysis and interpretation of the data, drafted the manuscript and contributed to the experimental design. EOF was involved in the design of experiments, interpretation of data, and preparation of the manuscript. MS and KS assisted in the preparation of reagents and in the interpretation of data and preparation of the manuscript.
